# Supplementary material for: Cerebral Venous-Associated Brain Damage May Lead to Anxiety and Depression
Source: J Clin Med. 2022 Nov 24;11(23):6927. doi: 10.3390/jcm11236927 (PMC9738348; doi:10.3390/jcm11236927)
Supplement: Supplementary file 1 [file jcm-11-06927-s001.zip › jcm-1897332-supplementary.pdf]

Supplementary Material

The detailed parameters of MRI

All participants underwent MRI sequence parameters with 3-tesla (MAGNETOM Verio, Siemens Healthcare, Erlangen, Germany) and a standard 32-channel head coil. Typical MRI sequence parameters included T1: repetition time (TR)/echo time (TE)=160/3.lms, field of view (FOV)=240×240mm^2; T2: TR/TE=3800/93ms, FOV=240×240mm^2; FLAIR: TR/TE=8000/94ms, FOV=218×240mm^2; DWI: TR/TE=5500/90ms, (FOV)=240×240mm^2.
